# Congenital lumbar vertebrae agenesis in a lamb

**Published:** 2017-12-15

**Authors:** Mohammad Farajli Abbasi, Bahador Shojaei, Omid Azari

**Affiliations:** 1 *Post Graduate Student of Veterinary Surgery, Shahid Bahonar University of Kerman, Kerman, Iran; *; 2 * Department of Basic Sciences, Faculty of Veterinary Medicine, Shahid Bahonar University of Kerman, Kerman, Iran; *; 3 * Department of Clinical Sciences, Faculty of Veterinary Medicine, Shahid Bahonar University of Kerman, Kerman, Iran.*

**Keywords:** Congenital anomaly. Lamb, Lumbar vertebrae agenesis

## Abstract

Congenital agenesis of lumbar vertebrae was diagnosed in a day-old female lamb based on radiology and clinical examinations. There was no neurological deficit in hindlimb and forelimb associated with standing disability. Radiography of the abdominal region revealed absence of lumbar vertebrae. Necropsy confirmed clinical and radiographic results. No other anomaly or agenesis was seen macroscopically in the abdominal and thoracic regions as well as vertebral column. Partial absence of vertebral column has been reported in human and different animal species, as an independent occurrence or associated with other organs anomalies. The latter has been designated as caudal regression syndrome. Vertebral agenesis may arise from irregularity in the differentiation of somites to the sclerotome or sclerotome to the vertebral primordium. Most of the previously reported cases of agenesis were related to the lumbosacral region, lonely or along with other visceral absences. This case was the first report of congenital agenesis of lumbar vertebrae in a lamb.

## Introduction

In the embryonic period, somites have a critical role in vertebral column formation. Somites will form from the paraxial mesoderm and fashion craniocaudally according to the cervical, thoracic, lumbar, sacral and caudal regions. Each somite differentiates into two cell groups of dermomyotome and sclerotome. Sclerotomes develop to vertebrae and the dermomyotome cells form muscle and overlying dermal tissue.^1^^,^^2^ Each half of a vertebra develops separately and then fuses with its counterpart, so capable of surrounding the notochord and spinal cord.^2^ Therefore, any disorder in the sclerotome differentiation affects the formation of the vertebral column and results in related anomalies.^1^^,^^3^

Most of the reported cases of vertebral anomalies of human and animals are according to the disturbance in the fusion process of ossification centers^4^^,^^5^ or failure of sclerotome differentiation.^2^^,^^6^^,^^7^ Among these, associated anomalies which occur in the visceral organs, soft tissues and neural tubes have been repeatedly reported.^3^^,^^8^ Lack of several vertebrae mostly occurs in the lumbosacral region of the vertebral column, however, it is usually accompanied by other defects which have been mentioned as caudal regression syndrome.^3^

We could not find any reported case of lumbar agenesis without any other defects in animal species and human. We aimed to describe the first one which was observed in a lamb.

## Case Description

**Fig. 1:**
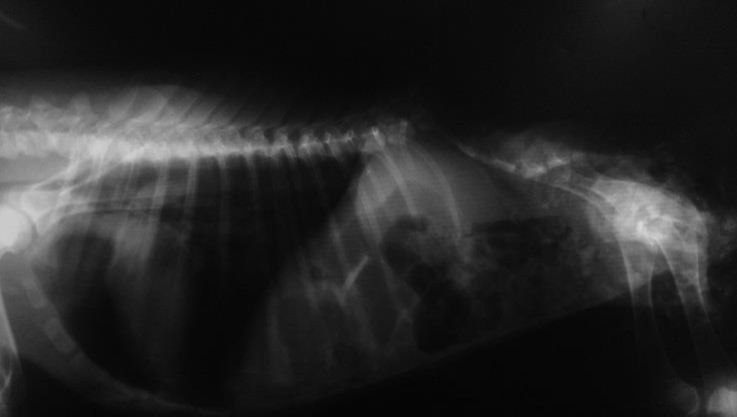
Lumbar vertebrae were absent, sacrum and pelvis were normal in the lateral radiograph.

**Fig. 2:**
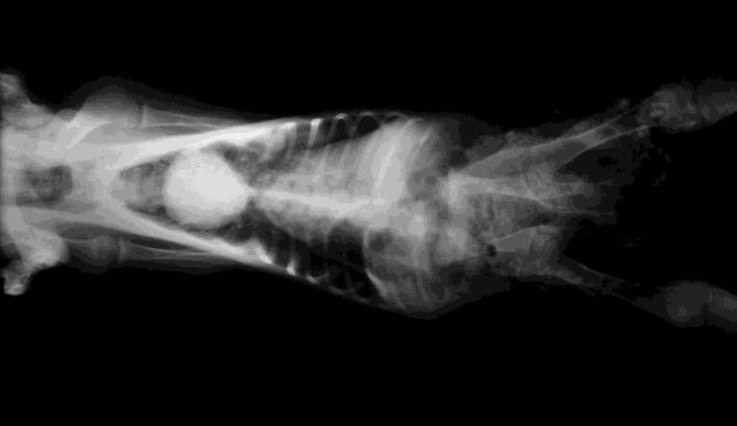
Thoracic and abdominal organs were seen normal, the sacrum and hind limbs were normal in venterodorsal radiograph.

A day-old female lamb, delivered as a single lamb of 5^th^ pregnancy of a healthy ewe, was presented to veterinary hospital, Shahid Bahonar University of Kerman, Kerman, Iran. Clinical examinations revealed normal body condition, muscle tonicity and integument. Both thoracic and pelvic limbs showed positive sensory reactions and there was no neurological deficit or cutaneous stigmata. The lamb had attempts for standing with no success. On whole body palpation, the lumbar vertebral anomaly was diagnosed. For further evaluation, the lamb was referred to diagnostic imaging section. Radiography of the abdominal region revealed that no lumbar vertebrae were seen in their place (Figs. 1 and 2). Blood biochemistry analysis was not performed. No other congenital anomaly was reported in the previous parturitions of the ewe and in the flock. 

**Fig. 3:**
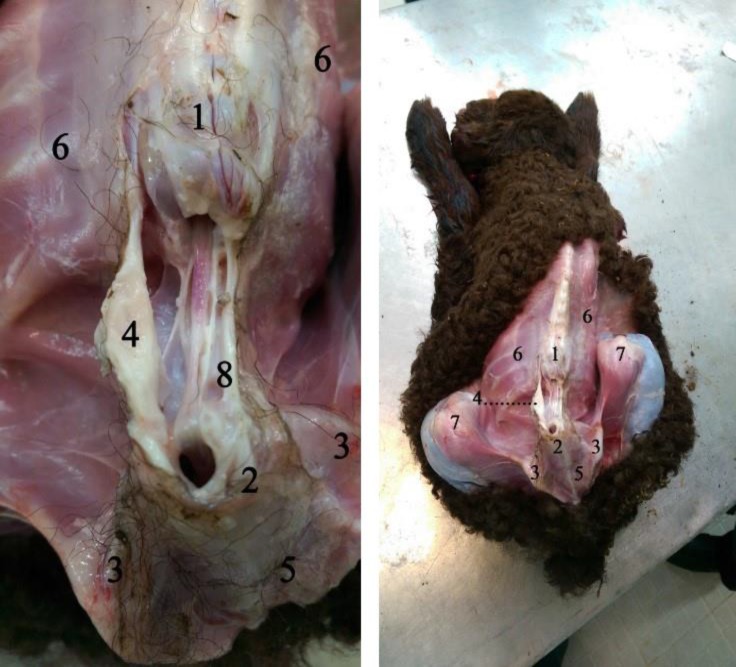
As seen in the photo that taken after autopsy, the spine was seem normal. 1) Thoracic vertebrae, 2) Sacrum, 3) Wing of ilium, 4) Muscular sheath, 5) Sacroiliac junction, 6) Ribs, 7) Stifle, 8) Spinal cord.

Vertebral column was normal in all other cervical, thoracic and sacral regions. Necropsy confirmed radiological findings (Fig. 3). No osseous or cartilaginous tissue was found in the lumbar region. The spinal cord was intact and detected in its right place without any macroscopic defect. 

It was the 5^th^ pregnancy of mother of presented lamb and there was not any problem or any kind of congenital anomaly in previous parturitions. Feeding of the herd was free grazing and all the lambs were completely healthy.

## Discussion

Defects in sclerotome differentiation can result in the anomalous development of the vertebral column. These defects have usually been categorized as spina bifida occulta, fusion of adjacent vertebrae and hemivertebrae.^9^ The first two defects result from disturbance in the fusion process of ossification centers. However, the hemivertebrae, the condition which is usually confined to the thoracolumbar region, is due to failure of sclerotome differentiation on one side of the developing vertebral body.^7^ In the present case, no lumbar vertebrae and other bony elements were diagnosed in the lumbar region. It was interesting that the musculature and the skin of the lumbar region were seen normal. It means that among the derivative cells of the somites of this region, only two groups of dermatome and myotome, have been differentiated and reached to their fate. In other words, while the sclerotome of the lumbar region has been affected, the other somitic cells of this region and the entire somites of the other regions showed no irregularity. 

Partial agenesis of vertebral column has been reported in the human and animals.^4^^,^^7^^,^^10^ Most of these cases describe sacral or sacrolumbar agenesis, alone or along with other soft tissue anomalies. According to Renshaw classification, spectrum of sacral agenesis based on bone and articular defect is classified into five types. Type I: unilateral total or partial agenesis of sacrum; type II: agenesis of variable lumbar and total sacrum and iliac articulate with the lowest vertebra sides; type III: agenesis of variable lumbar and total sacrum, and the caudal end plate of the lowest vertebra rests fusion with iliac or an iliac amphiarthrosis; type IV: both lower limb soft tissue fusing and type V: formation a single femur and tibia, also known as sirenomelia or mermaid syndrome.^3,7^ In the present case, only lumbar vertebral agenesis without any other anomaly in the vertebral column or adjacent soft tissues was observed. It could be attributed to the disorder of the sclerotomal cells and formation or differentiation in the lumbar region.

Lack of lumbar vertebrae has been reported as an associated anomaly in the type II and III of sacral agenesis^5^^,^^7^^,^^11^ and renal agenesis^7^ in the human and animals. However, according to the literature this was the first report of lumbar vertebrae agenesis without any other mentioned anomalies.
